# Early BMI-associated increases in central and peripheral blood pressure in primary school children: evidence of a continuous, nonlinear relationship below the overweight threshold

**DOI:** 10.1007/s00431-026-07584-0

**Published:** 2026-10-29

**Authors:** Jeannine von der Born, Lisa Wilmsmeier, Rizky I. Sugianto, Carl Grabitz, Tim Alexander Ubenauf, Elena Lehmann, Nima Memaran, Ana Céline Braun, Denise Homeyer, Uwe Tegtbur, Bernhard Magnus Wilhelm Schmidt, Anette Melk

**Affiliations:** 1 Department of Paediatric Kidney, Liver and Metabolic Diseases and Neuropaediatrics, Hannover Medical School, Carl-Neuberg-Str. 1, 30625 Hannover, Germany; 2 Clinic for Rehabilitation Medicine and Sport Medicine, Hannover Medical School, Carl-Neuberg-Str. 1, 30625 Hannover, Germany; 3 Department of Nephrology and Hypertension, Hannover Medical School, Carl-Neuberg-Str. 1, 30625 Hannover, Germany

**Keywords:** Blood pressure, Central blood pressure, Body mass index, Children, Cardiovascular risk

## Abstract

Childhood overweight is associated with elevated blood pressure (BP) and future cardiovascular risk. We aimed to determine the body mass index percentile (BMIp) at which blood pressure (BP) begins to increase in children, particularly below the overweight threshold. Central BP may provide additional insight into early hemodynamic changes. In a cohort of primary school children, peripheral and central BP were measured using standardized protocols. Associations of BMIp with BP were evaluated using spline and linear regression models adjusted for age, sex, and height. BMIp categories were used to identify thresholds at which BP was significantly higher than in children with BMIp < 50. Among 1,183 children (mean age 7.9 years), BP increased progressively with rising BMIp, with the greatest elevations in children with overweight or obesity. Significant increases in peripheral and central systolic BP were observed at BMIp levels below the WHO-defined cutoff for overweight, central BP increased at lower BMIp than peripheral BP. Although peripheral diastolic BP was not associated with BMIp, central diastolic BP increased significantly across higher BMIp ranges.

*Conclusions*: In primary school children, BMI-associated increases in BP are detectable at substantially lower BMIp than commonly considered, reflecting a continuous nonlinear relationship. Both peripheral and central BP increased within the high-normal BMI-range. Central BP identified early, distinct BP alterations, including significant diastolic changes not detected with peripheral BP. These findings suggest that cardiovascular risk related to body weight arises along a continuum and support the potential value of early BP monitoring in children with increasing BMI.

*Trial registration*: Trial Registration Number: NCT04942431. Date of Registration: 08.04.2021.

**Table:** 

**What is Known** • *Childhood overweight is associated with elevated blood pressure, both are key risk factors for future cardiovascular disease*. • *However, the body mass index level at which blood pressure begins to rise in children remains unclear.*
**What is New** • *We identify specific body mass index ranges at which blood pressure increased significantly, highlighting children with high-normal body weight as a group at early cardiovascular risk.*

**Graphical Abstract:**

**Figure:**
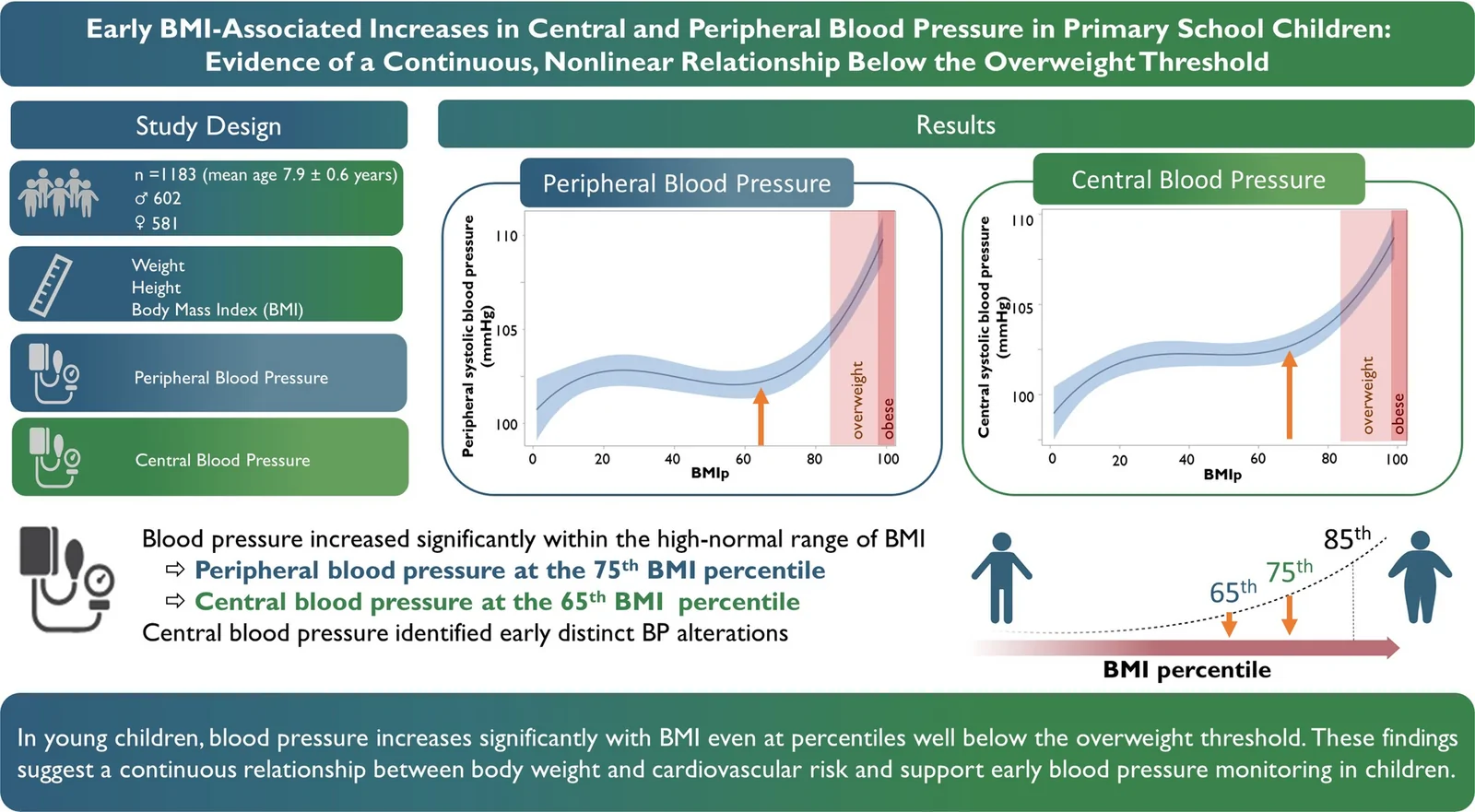

**Supplementary Information:**

The online version contains supplementary material available at https://doi.org/10.1007/s00431-026-07584-0.

## Introduction

The global prevalence of overweight and obesity in children and adolescents has increased markedly over recent decades and remains high [1, 2]. Excess body weight during childhood is strongly associated with elevated blood pressure (BP) and the development of arterial hypertension [3, 4], increasing the risk of hypertension and cardiovascular disease in adulthood [5, 6]. BP levels track from childhood into adult life, and elevated BP in childhood is among the strongest predictors of later hypertension [5]. Importantly, early vascular alterations and signs of cardiovascular dysfunction, which are considered precursors of overt cardiovascular disease, have been observed even before puberty, linked to both elevated BP [7] and increased body weight [8]. These findings underscore the clinical relevance of identifying early determinants of BP elevation during childhood.

Despite this well-established association between body weight and BP, it remains unclear at which body weight level BP begins to increase in children. In pediatric practice, overweight and obesity are currently defined using body mass index percentiles (BMIp) derived primarily from statistical distributions rather than health outcomes or the emergence of cardiovascular risk [9]. Clinically apparent obesity-related comorbidities are rare in childhood and usually limited to severe obesity [8]. However, metabolic alterations and early cardiovascular changes occur much earlier along the continuum of increasing body weight [10]. Consequently, the BMIp at which BP, and thereby cardiovascular risk, starts to rise remains uncertain, particularly in children who are still classified as normal weight.

Hypertension represents one of the most common and modifiable cardiovascular risk factors, contributing substantially to global cardiovascular morbidity and mortality [11]. From a preventive perspective, understanding when and under which conditions BP begins to rise in childhood is essential. Even small BP differences in childhood are considered clinically meaningful, as they tend to persist over time and may amplify cardiovascular risk through cumulative exposure [12]. Identifying early BP increases below established overweight thresholds could therefore have important implications for pediatric risk stratification, parental counseling, and early lifestyle interventions.

In adults, central BP is well established and provides clinically relevant information beyond peripheral BP [13]. In children, its clinical relevance remains debated, partly due to pronounced age-related pressure amplification [14]. Nevertheless, emerging evidence suggests central BP may improve the identification of arterial hypertension and early cardiovascular target organ damage in high-risk pediatric populations [15–17]. Whether central BP increases earlier or more sensitively than peripheral BP with increasing body weight in young children has not yet been systematically investigated.

We hypothesized that significant increases in BP occur at BMIp levels below the currently accepted threshold for overweight in children and adolescents. The aim of this study was therefore to identify the BMI percentile at which peripheral and central BP increase significantly in a large cohort of healthy primary school children.

## Methods

### Study design and cohort

All primary schools in the regions of Hanover and Wolfsburg, Germany, were approached with the support of the Ministry of Education of Lower Saxony. Thirty-three schools agreed to participate in the observational longitudinal “Rebirth Active School 2.0” trial. This analysis used cross-sectional baseline data collected between June 2021 and July 2023. Written informed consent was obtained from 1,247 children and their parents. After excluding 64 children due to incomplete data or withdrawal, 1,183 first- and second-grade children were included. The study followed the Declaration of Helsinki and was approved by the Ethics Committee of Hannover Medical School (No. 7290).

#### Anthropometry

Weight, height, and waist circumference were measured according to standardized procedures [18]. BMI was calculated as weight divided by height squared (kg/m^2^). For better comparability, age- and sex-specific z-scores for height, weight, and BMI (BMIz) were calculated [19–21]. In the subsequent analyses, BMIp was used. Overweight was defined as BMIp ≥ 84.1th to < 97.7th (BMIz ≥ 1.0 and < 2.0), and obesity as BMIp ≥ 97.7th (BMIz ≥ 2.0) [20]. A BMIp > 60th but still below the overweight threshold was defined as high normal body weight [22].

### Blood pressure measurements

Peripheral BP was measured using a validated oscillometric device (Dinamap VC150, GE Healthcare, Chicago, Illinois, USA) following current guidelines [14]. In brief, children were seated and rested for five minutes before three measurements on each arm, spaced 1–2 min apart. The mean of the second and third right-arm measurements was used for analysis. BP values were expressed as z-scores standardized for sex, age, and height [23] and classified according to BP percentiles. High-normal BP (pre-hypertension) was defined as peripheral systolic blood pressure (pSBP) and/or peripheral diastolic blood pressure (pDBP) ≥ 90th percentile and < 95th percentile (z-score ≥ 1.28 and < 1.65). Hypertension was defined as peripheral systolic BP and/or peripheral diastolic BP ≥ 95th percentile for sex, age, and height (z-score ≥ 1.65) [14].

Central BP was estimated using the Mobil-O-Graph device (I.E.M. GmbH, Stollberg, Germany), a validated oscillometric system [24, 25]. Measurements were performed with the child in a 30° supine position. Three measurements were obtained on the right arm after five minutes' rest. The device software, the ARC Solver application (Austrian Institute of Technology, Vienna, Austria), allows the estimation of central BP using different calibration approaches. Calibration method C2 is based on mean arterial pressure and peripheral diastolic BP. Previous data from our group showed that central BP (calibration method C2) is superior to peripheral BP in predicting left ventricular mass index and pulse wave velocity, both established markers of cardiovascular target organ damage [15]. Accordingly, all analyses were performed using central BP values obtained with calibration method C2, with the mean of the last two measurements of acceptable signal quality used for analysis [24]. Due to technical limitations, central BP C2 values could not be obtained for all children. Measurements were included in the analysis only when technically valid, high-quality waveforms were obtained. Reference values for calibration method C2 are currently not available in children, and therefore z-scores could not be calculated.

### Statistical analysis

Data are presented as mean ± standard deviation (SD) or as absolute numbers and percentages. Normality of continuous variables was assessed using the Shapiro–Wilk test. As a first approach, spline regression analyses were performed to detect potential nonlinear relationships between BMI and peripheral or central BP.

For further assessment and improved visualization, linear regression models were applied to predict BP values for children in the following BMIp categories: ≥ 50th, ≥ 60th, ≥ 70th, ≥ 80th, and ≥ 90th. All models were adjusted for age, sex, and height. Children below the 50th BMIp served as a fixed reference group that was compared separately with each BMI category, which included all children at or above the respective BMIp threshold.

Because increases in BP are likely driven by children with higher BMIp, and in order to further specify the BMIp at which BP increased significantly compared with children with a BMIp < 50th, we additionally defined mutually exclusive BMIp categories (≥ 50th- < 65th, ≥ 65th- < 75th, ≥ 75th- < 85th, ≥ 85th- < 95th, and ≥ 95th). In contrast to the first approach, these BMI categories did not overlap. Linear regression models adjusted for age, sex, and height were used to compare the corrected means of BP for each BMIp group with those for the reference group (< 50th BMIp).

A two-sided *p*-value < 0.05 was considered statistically significant. All statistical analyses were performed using SAS Enterprise Guide version 7.1 (Statistical Analysis Software, Cary, North Carolina, USA).

## Results

### Patient characteristics

A total of 1,183 children were included in the analysis, mean age was 7.9 ± 0.6 years (range 6.3–10.4 years), 49.1% (*n* = 581) were girls. Mean height was 129.8 ± 6.5 cm (z-score 0.5 ± 1.0), and mean BMI at 16.7 ± 2.8 kg/m^2^ (z-score 0.1 ± 1.1); BMIz was normally distributed. Overall, 18.4% (*n* = 218) of the children were classified as overweight and 5.1% (*n* = 60) as obese. Further demographic and anthropometric characteristics are summarized in Table 1.

**Table 1:** Demographic and anthropometric characteristics of the study population (*n* = 1,183)

	Mean ± SD or absolute number (percentage/range)
Age [years]	7.9 ± 0.6 (6.3–10.4)
Sex, male/female	602 (50.9%)/581 (49.1%)
Height	
absolute [cm]	129.8 ± 6.5 (112.1–151.5)
z-score	0.5 ± 1.0 (− 2.9–3.95)
Weight	
absolute [kg]	28.4 ± 6.6 (16.7–65.2)
z-score	0.4 ± 1.1 (− 3.2–3.2)
BMI	
absolute [kg/m^2^]	16.7 ± 2.8 (12.1–31.6)
z-score	0.1 ± 1.1 (− 4.1–2.8)
BMIp < 50th	543 (45.9%)
BMIp ≥ 50th— < 65th	158 (13.4%)
BMIp ≥ 65th— < 75th	97 (8.2%)
BMIp ≥ 75th— < 85th	116 (9.8%)
BMIp ≥ 85th— < 95th	147 (12.4%)
BMIp ≥ 95th	122 (10.3%)

Abbreviations: *BMI* body mass index, *BMIp* body mass index percentile, *SD* standard deviation

Absolute and standardized BP values are summarized in Table 2. Peripheral blood pressure: Mean peripheral systolic BP was 103.8 ± 8.3 mmHg, and mean peripheral diastolic BP was 61.2 ± 5.7 mmHg. High-normal peripheral systolic and/or peripheral diastolic BP values were observed in 111 children (9.4%), while 97 children (8.2%) had peripheral systolic BP and/or peripheral diastolic BP values within the hypertensive range. Central blood pressure: Mean central systolic blood pressure (cSBP) and central diastolic blood pressure (cDBP) were 103.2 ± 7.4 mmHg and 65.3 ± 6.0 mmHg, respectively (Table 2).

**Table 2:** Peripheral and central BP values of the study population, expressed as absolute values and z-scores

	Mean ± SD (range) or absolute number (frequency)
Peripheral systolic BP (*n* = 1,183)	
absolute [mmHg]	103.8 ± 8.3 (81.0–144.0)
z-score	0.6 ± 0.8 (− 1.4–4.3)
≥ 90th pct— < 95^th^pct	*n* = 100 (8.5%)
≥ 95th pct	*n* = 94 (8.0%)
Peripheral diastolic BP (*n* = 1,183)	
absolute [mmHg]	61.2 ± 5.7 (48.5–88.5)
z-score	0.3 ± 0.5 (− 0.9–2.4)
≥ 90th pct— < 95^th^pct	*n* = 30 (2.5%)
≥ 95th pct	*n* = 11 (0.9%)
Prevalence of pre-hypertensive and hypertensive BP values	
pBP ≥ 90th— < 95th pct	*n* = 111 (9.4%)
pBP ≥ 95th pct	*n* = 97 (8.2%)
Central systolic BP (*n* = 1,117)	
absolute [mmHg]	103.2 ± 7.4 (81.0–134.5)
Central diastolic BP (*n* = 1,117)	
absolute [mmHg]	65.3 ± 6.0 (47.0–88.5)

Pre-hypertension was defined as peripheral systolic BP and/or peripheral diastolic BP ≥ 90th percentile and < 95th percentile (z-score ≥ 1.28 and < 1.65). BP values were considered to be within the hypertensive range if peripheral systolic BP and/or peripheral diastolic BP was ≥ 95th percentile for sex, age, and height (z-score ≥ 1.65) [14]

Abbreviations: *BP* blood pressure, *pct* percentile, *SD* standard deviation

### Prevalence of elevated BP according to children’s BMI

Considering only normal weight children with a BMIp < 85th, 84.8% of the study population had normal peripheral BP values, 8.4% had high-normal BP, and 6.8% had BP values within the hypertensive range. In children classified as overweight, 12.8% exhibited high-normal BP and 9.6% had BP values within the hypertensive range. In children classified as obese, 11.7% showed high-normal BP, while 26.7% had peripheral BP values within the hypertensive range.

**Fig. 1:**
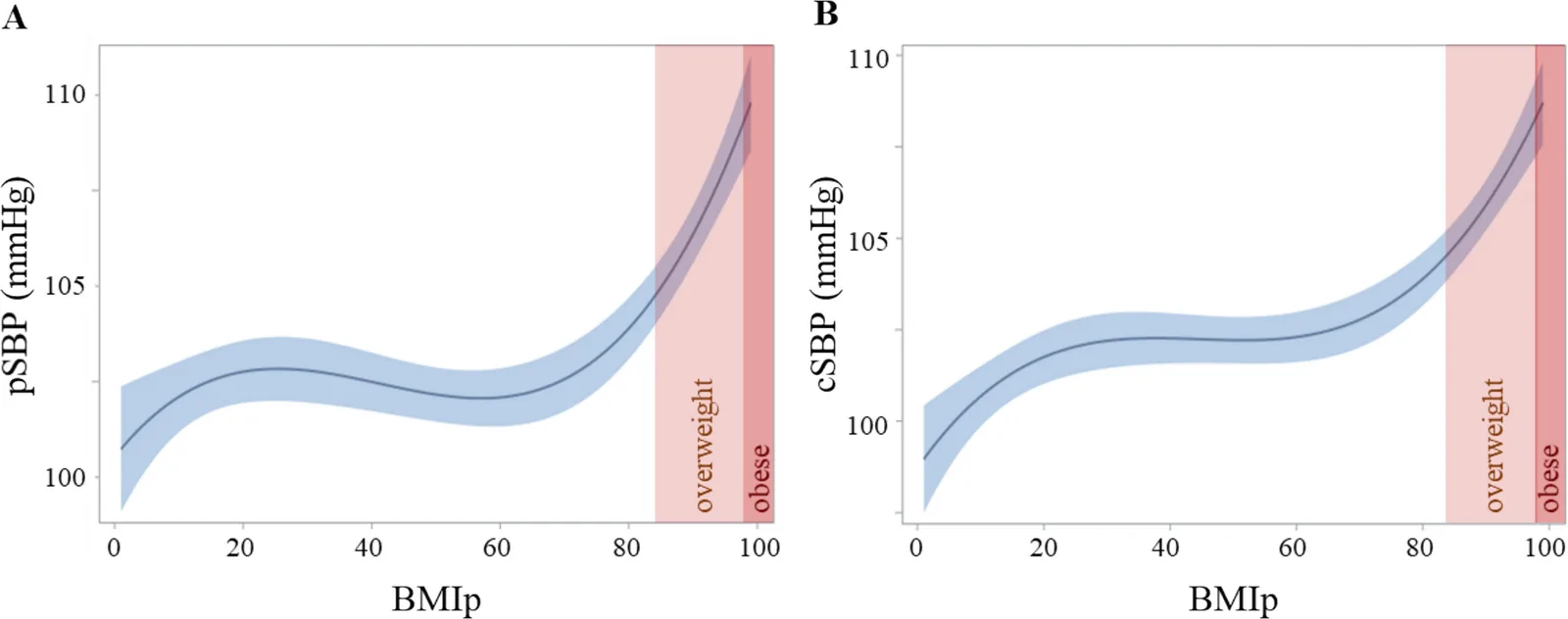
Early nonlinear increases in systolic BP across the BMI percentile spectrum. Spline regression analyses illustrating nonlinear associations between BMIp and systolic BP. Panels show (**A**) peripheral systolic BP and (**B**) central systolic BP. BP values begin to increase at approximately the 60th BMIp, with steeper slopes observed at higher BMIp levels. Abbreviations: BMIp, body mass index percentile; BP, blood pressure; pct, percentile; pSBP, peripheral systolic blood pressure; cSBP, central systolic blood pressure

As higher BMI in children is known to be associated with higher BP values, we evaluated the effect of increasing BMIp on peripheral and central BP in our cohort of primary school children. In a first approach, spline regression analyses were performed to explore potential nonlinear associations between BMIp and peripheral as well as central BP. The analyses demonstrated an increase in peripheral systolic BP as well as in central systolic BP starting approximately at the 60th BMIp, with a markedly steeper slope at higher BMIp levels (≥ 85th) (Fig. 1A, B). Comparable patterns were observed for diastolic BP, with increases in peripheral diastolic BP and central diastolic BP also becoming apparent approximately at the 60th BMIp (Fig. S1A, B).

**Fig. 2:**
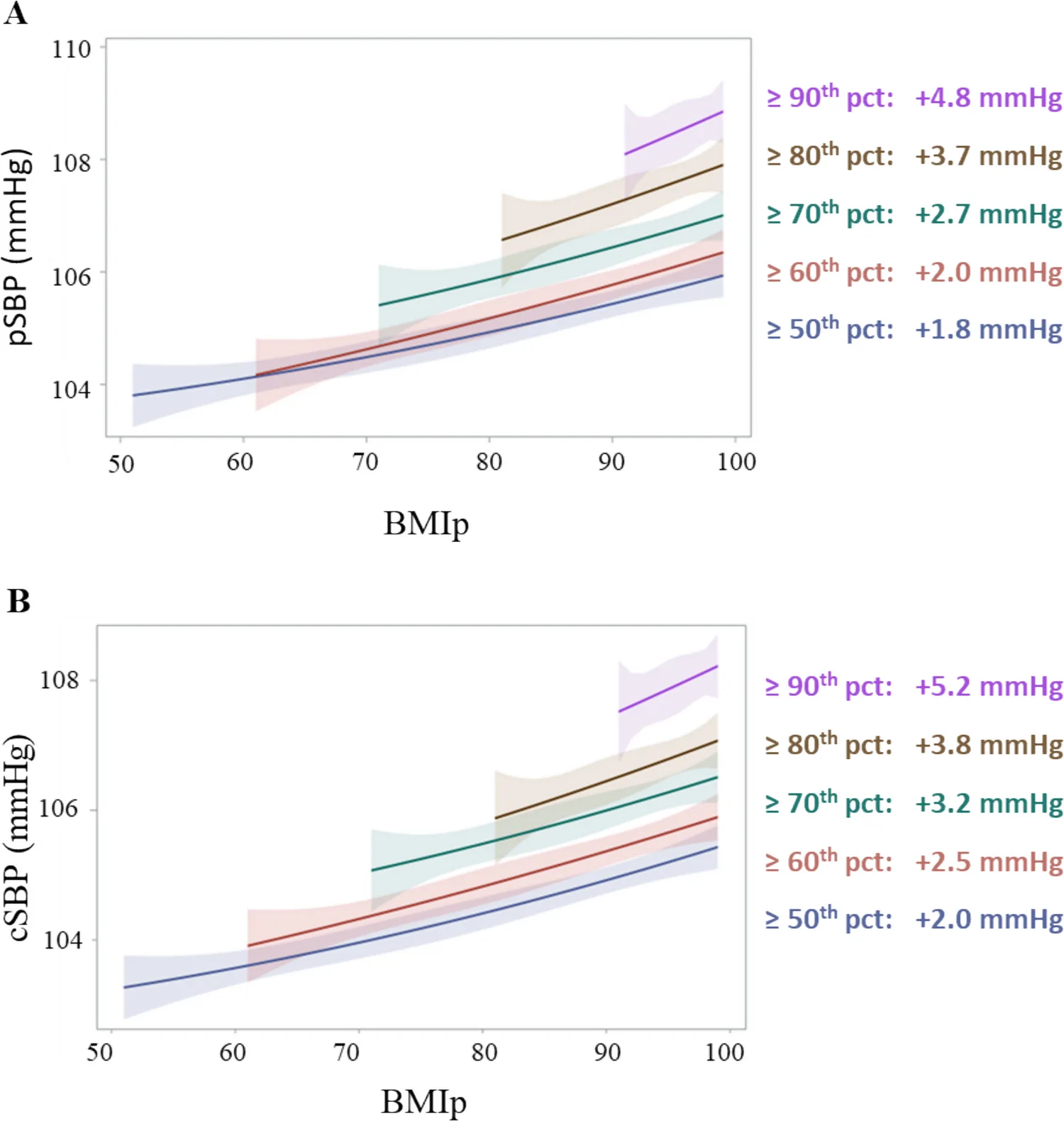
Progressive increases in systolic BP across increasing BMI percentile categories. Panels show predicted mean values for (**A**) peripheral systolic BP and (**B**) central systolic BP across increasing BMIp categories. All models demonstrated significant increases in systolic BP with increasing BMIp. Linear regression models adjusted for age, sex, and height were used to estimate BP for each BMIp category (≥ 50th, ≥ 60th, ≥ 70th, ≥ 80th, and ≥ 90th), with children with BMIp < 50th serving as the reference group. Abbreviations: BMIp, body mass index percentile; BP, blood pressure; pct, percentile; pSBP, peripheral systolic blood pressure; cSBP, central systolic blood pressure

In a next step, linear regression models were applied. Children with a BMIp < 50th served as the reference group and were compared with five partially overlapping BMIp categories (BMIp ≥ 50th, ≥ 60th, ≥ 70th, ≥ 80th, and ≥ 90th). These models revealed significant increases in peripheral systolic BP and central systolic BP with increasing BMIp, already detectable at BMIp ≥ 50th (Fig. 2A, B). The estimates for peripheral systolic BP increased with each BMIp category, from 1.8 mmHg (95% CI 0.8–2.7) for children with BMIp ≥ 50th to 4.8 mmHg (95% CI 3.4–6.2) for children with BMIp ≥ 90th, compared with the reference group (Fig. 2A). Estimates for central systolic BP were of comparable magnitude (Fig. 2B). While there was no significant association between BMIp and peripheral diastolic BP (Fig. S2A), central diastolic BP increased significantly across all BMIp categories, starting at BMIp ≥ 50th (Fig. S2B).

Assuming that the early and significant increases in BP observed in the previous analyses were primarily driven by children with higher BMIp levels, five mutually exclusive, non-overlapping BMIp categories were defined (Tables 3 and S1). This approach ensured that children with higher BMIp levels did not influence BP estimates in lower BMIp categories. For peripheral systolic BP, the first statistically significant increase was observed with a BMIp ≥ 75th and < 85th (β = 1.8 mmHg, *p* = 0.03), indicating that children within this BMIp range already exhibited higher peripheral systolic BP compared with the reference group. For central systolic BP, significant increases were detected at even lower BMIp levels (≥ 65th- < 75th:β = 1.6 mmHg, *p* = 0.04). Systolic BP further increased between the 85th and 95th BMIp (pSBP: β = 3.13 mmHg; cSBP: β = 3.39 mmHg). In children with a BMIp ≥ 95th, systolic BP increases were most pronounced, with peripheral systolic BP and central systolic BP values exceeding those of the reference group by more than 5 mmHg (pSBP: β = 5.48 mmHg; cSBP: β = 5.20 mmHg). In contrast to peripheral diastolic BP, which showed no significant association with BMIp, central diastolic BP increased significantly across BMIp categories starting at BMIp ≥ 75th (β = 1.56 mmHg, *p* = 0.01) (Table S1). Overall, the increases in central diastolic BP with increasing BMIp were less pronounced compared to central systolic BP.

**Table 3:** Association of increasing BMI percentile with peripheral and central systolic BP

	Peripheral systolic BP		Central systolic BP	
	*n* = 1,183		*n* = 1,117	
	β	*p*-value	β	*p*-value
Intercept	63.931	< 0.0001	63.631	< 0.0001
BMIp < 50th (reference)	0		0	
BMIp ≥ 50th- < 65th	−0.508	0.469	−0.064	0.920
BMIp ≥ 65th- < 75th	0.109	0.899	1.618	**0.035**
BMIp ≥ 75th- < 85th	1.792	**0.025**	1.601	**0.026**
BMIp ≥ 85th- < 95th	3.134	** < 0.0001**	3.388	** < 0.0001**
BMIp ≥ 95th	5.483	** < 0.0001**	5.201	** < 0.0001**
Sex (reference: male)	−1.178	0.009	−0.514	0.208
Age [years]	1.260	0.003	0.649	0.092
Height [cm]	0.227	< 0.0001	0.260	< 0.0001

Linear regression models for peripheral systolic BP and central systolic BP adjusted for age, sex, and height. Children with a BMIp < 50th served as the reference group and were compared with each of five mutually exclusive, non-overlapping BMIp categories. Statistically significant differences compared with the reference group are highlighted

Abbreviations: *BMIp* body mass index percentile, *BP* blood pressure

## Discussion

In this large cohort of healthy primary school children, we demonstrate that increases in BP occur at substantially lower BMIp levels than currently assumed. Notably, both peripheral and central BP already rised within the high-normal BMI range, well below the WHO-defined threshold for overweight. Central BP, in particular, showed significant increases at even lower BMIp than peripheral BP, suggesting a high sensitivity of central BP to weight-related cardiovascular changes. In addition, a differential pattern was observed for diastolic BP, with significant associations detectable only for central, but not peripheral, diastolic BP. These findings extend existing evidence linking childhood body weight and BP by identifying specific BMIp ranges at which BP increases significantly, thereby closing an important knowledge gap regarding early cardiovascular risk in children at substantially lower BMIp than commonly considered.

Our findings highlight the high-normal BMI range as a clinically relevant window for early BP increases in childhood. Several longitudinal studies have shown that children and adolescents with BMI values below the conventional overweight threshold already carry an increased risk for later hypertension. In a large population-based cohort of over 800,000 children and adolescents, high-normal BMI was associated with a significantly increased risk of incident hypertension within only a few years of follow-up [22]. These data are in line with our observation that BP increases are detectable well before children reach the WHO-defined overweight threshold, supporting the concept of cardiovascular risk emerging along a continuum rather than at discrete BMI cutoffs. Alternative definitions of childhood overweight have attempted to account for biological relevance rather than purely statistical distributions. The International Obesity Task Force (IOTF) proposed age- and sex-specific BMI cut-offs linked to adult BMI thresholds associated with cardiometabolic risk [26]. However, studies evaluating these cut-offs in pediatric populations demonstrated high specificity but limited sensitivity for identifying children with clustered cardiovascular risk factors, even when overweight was defined at relatively low BMIp [27]. Against this background, our data add important evidence that BP-related cardiovascular risk begins to increase already in children who are still classified as normal weight by current definitions, underscoring the need to better characterize risk within the high-normal BMI range.

Although the absolute BP differences observed in children with high-normal BMI were modest, they are likely clinically meaningful. BP levels are known to track from childhood into adulthood, and childhood BP is among the strongest predictors of adult hypertension [5, 28]. Even small early–life BP elevations may therefore translate into an increased lifetime exposure to elevated BP, thereby amplifying long-term cardiovascular risk. Differences as small as 1 mmHg are considered relevant at the population level [12], underscoring the clinical importance of 1–2 mmHg BP increases observed in children with high-normal BMI in our cohort. Moreover, nearly half of young adults with arterial hypertension had elevated BP already in childhood [5], highlighting the early origins of hypertensive disease. From this perspective, the pronounced BP differences in children with overweight and obesity, exceeding 5 mmHg compared with lean peers, are particularly concerning. Our findings support the concept that weight-associated BP elevations begin early in life and may accumulate over time, contributing to cardiovascular risk long before overt hypertension becomes clinically apparent.

Our findings suggest that central BP may provide additional insight into early weight-associated cardiovascular changes in children. In our cohort, central systolic BP increased significantly at lower BMIp levels than peripheral systolic BP. While pediatric reference values for C2-derived central blood pressure are currently unavailable, preventing classification as “normal” or “abnormal” in the same way as peripheral blood pressure, a difference of 1.6 mmHg in central systolic BP at the 65th– < 75th BMIp should not be dismissed as trivial. Given the strong tracking of BP from childhood to adulthood and the established link between even small increases in BP and early cardiovascular target organ damage, such differences may carry significant long-term cardiovascular implications, even in normotensive children [6, 29].

Peripheral and central BP were assessed using different measurement methods and in slightly different body positions and are therefore not directly comparable. Nevertheless, these differences support the hypothesis that there is a great sensitivity of central hemodynamic parameters to early weight-related vascular changes. This was particularly evident for diastolic BP: while peripheral diastolic BP did not change significantly across increasing BMIp levels, central diastolic BP showed a consistent and significant increase. This observation is physiologically plausible, as central BP more directly reflects the load imposed on central arteries and target organs than peripheral measurements [30]. Previous pediatric studies have shown that elevated central BP is more closely associated with subclinical cardiovascular target organ damage than peripheral BP, including markers of arterial stiffness and vascular remodeling [15–17]. Moreover, in high-risk pediatric populations, central BP improves detection of uncontrolled or previously unrecognized hypertension [15]. Although not yet part of routine pediatric guidelines, our data support using central BP to complement risk stratification in children with increasing body weight or early weight gain.

Several limitations of this study should be acknowledged. The cross-sectional design precludes any causal inference between BMIp and BP. BP was assessed during a single study visit, which does not allow a formal diagnosis of arterial hypertension and may have resulted in transient BP elevations due to situational effects. The significant differences of 1–2 mmHg observed in the lower BMI ranges should be interpreted as population-level associations rather than evidence of sustained BP elevation in individual children. Ambulatory BP monitoring was not feasible in the school setting. Residual confounding by unmeasured factors, including pubertal development, socioeconomic status, lifestyle-related factors, and missing central blood pressure measurements in some children, cannot be excluded. Pubertal status was not assessed; however, given the young age of the cohort, it is likely that most children were prepubertal. Finally, markers of cardiovascular target organ damage were not available, precluding direct conclusions regarding the clinical significance of the observed BP differences.

Taken together, our findings have important implications for pediatric cardiovascular prevention. Children with increasing body weight, even within the high-normal BMI range, may benefit from closer monitoring, including regular BP assessment, to identify early cardiovascular risk. Given the strong tracking of BP into adulthood, early identification of subtle BP elevations may provide a window for timely lifestyle interventions. In this context, the assessment of central BP may offer complementary information, particularly for identifying early alterations in diastolic BP that are not apparent from peripheral BP measurements. Evidence from interventional studies indicates that weight reduction and lifestyle modification in prepubertal children can not only lead to improvements in BP but also to favorable changes in vascular structure and function, underscoring the potential for early prevention to modify long-term cardiovascular trajectories [31–35]. Beyond individual-level prevention, our results also contribute to the ongoing discussion on how overweight and obesity should be defined in childhood. Large adult cohorts have shown that BMI cut-offs derived from cardiovascular risk rather than statistical distributions are feasible and may improve risk stratification [36]. While such approaches cannot be directly transferred to children, our data support the need for longitudinal pediatric studies integrating BMI trajectories, BP development, and markers of cardiovascular target organ damage. Such studies will be essential to determine whether risk-based BMI thresholds could complement existing definitions and ultimately improve early cardiovascular prevention in children and adolescents.

## Supplementary Information

Below is the link to the electronic supplementary material.

Supplementary file1 (DOCX 255 KB)

## Data Availability

The datasets generated and analyzed during the study are available from the corresponding author upon reasonable request.

## Non-standard abbreviations and Acronyms

BMI: Body mass index
BMIp: Body mass index percentile
BMIz: Body mass index z-score
BP: Blood pressure
cDBP: Central diastolic blood pressure
cSBP: Central systolic blood pressure
pDBP: Peripheral diastolic blood pressure
pSBP: Peripheral systolic blood pressure
SD: Standard deviation
WHO: World Health Organization
