# Supplementary material for: Early BMI-associated increases in central and peripheral blood pressure in primary school children: evidence of a continuous, nonlinear relationship below the overweight threshold
Source: Eur J Pediatr. 2026 Oct 29;185(11):907. doi: 10.1007/s00431-026-07584-0 (PMC13719791; doi:10.1007/s00431-026-07584-0)
Supplement: Supplementary file 1 [file 431_2026_7584_MOESM1_ESM.docx]

**Supplemental Material to**

**Early BMI-Associated Increases in Central and Peripheral Blood Pressure**

**in Primary School Children: Evidence of a Continuous, Nonlinear Relationship Below the Overweight Threshold**

Jeannine von der Born MD^1*^; Lisa Wilmsmeier^1*^, Rizky I. Sugianto DDS, PhD^1^; Carl Grabitz MD^1^; Tim Alexander Ubenauf^1^, Elena Lehmann MSc^1^; Nima Memaran MD^1^; Ana Céline Braun^2^; Denise Homeyer^2^, Uwe Tegtbur MD^2^, Bernhard Magnus Wilhelm Schmidt MD, MSc^3^; Anette Melk MD, PhD^1^

^1^Department of Paediatric Kidney, Liver and Metabolic Diseases and Neuropaediatrics, Hannover Medical School, Carl-Neuberg-Str. 1, 30625 Hannover, Germany

^2^Clinic for Rehabilitation Medicine and Sport Medicine, Hannover Medical School, Carl-Neuberg-Str. 1, 30625 Hannover, Germany

³Department of Nephrology and Hypertension, Hannover Medical School, Carl-Neuberg-Str. 1, 30625 Hannover, Germany

*Jeannine von der Born and Lisa Wilmsmeier contributed equally

**Short title:** Early BMI-Associated BP Increases in Children

Jeannine von der Born ORCID: 0009-0007-5599-8604

Lisa Wilmsmeier ORCID: 0009-0006-9084-4478

**Corresponding author:**

Anette Melk MD, PhD

Department of Paediatric Kidney, Liver and Metabolic Diseases and Neuropaediatrics, Hannover Medical School

Carl-Neuberg-Str. 1; 30625 Hannover; Germany

Email: melk.anette@mh-hannover.de

ORCID: 0000-0002-8164-6318

**Table S1 Association of increasing BMI percentile with peripheral and central diastolic BP.**

**Linear regression models for peripheral diastolic BP and central diastolic BP adjusted for age, sex and height. Children with a BMIp <50^th^ served as the reference group and were compared with each of five BMIp categories. Statistically significant differences compared with the reference group are highlighted.**

|  | Peripheral diastolic BP | | Central diastolic BP | |
| --- | --- | --- | --- | --- |
|  | n= 1,183 | | n=1,117 | |
|  | **β** | **p-value** | **β** | **p-value** |
| Intercept | 45.094 | <0.0001 | 46.497 | <0.0001 |
| BMIp <50^th^ (reference) | 0 |  | 0 |  |
| BMIp ≥50^th^-<65^th^ | -0.931 | 0.068 | -0.245 | 0.652 |
| BMIp ≥65^th^-<75^th^ | -0.416 | 0.502 | 1.037 | 0.113 |
| BMIp ≥75^th^-<85^th^ | 0.139 | 0.811 | 1.556 | **0.011** |
| BMIp ≥85^th^-<95^th^ | 0.067 | 0.901 | 2.214 | **0.0001** |
| BMIp ≥95^th^ | 0.647 | 0.278 | 2.328 | **0.0003** |
| Sex (reference: male) | -0.012 | 0.972 | -0.247 | 0.478 |
| Age [years] | 0.581 | 0.060 | 0.756 | 0.022 |
| Height [cm] | 0.090 | 0.004 | 0.094 | 0.005 |
| *Abbreviations: BMIp, body mass index percentile; BP, blood pressure.* | | | | |

**Fig. S1 Nonlinear associations between BMI percentile and diastolic BP.**

Spline regression analyses illustrating nonlinear associations between BMIp and diastolic BP. Panels show (A) peripheral diastolic BP and (B) central diastolic BP. Diastolic BP increases become apparent at approximately the 60^th^ BMIp, with steeper slopes at higher BMIp levels.

*Abbreviations: BMIp, body mass index percentile; BP, blood pressure; pDBP, peripheral diastolic blood pressure; cDBP, central diastolic blood pressure.*

**Fig. S2 Divergent associations of BMI percentile with peripheral and central diastolic BP.**

Panels show predicted mean values for (A) peripheral diastolic BP and (B) central diastolic BP across increasing BMIp categories. No significant association was observed between BMIp category and peripheral diastolic BP, whereas central diastolic BP increased significantly across BMIp categories.

Linear regression models adjusted for age, sex, and height were used to estimate BP for each BMIp category (≥50^th^, ≥60^th^, ≥70^th^, ≥80^th^, and ≥90^th^), with children with BMIp <50^th^ serving as the reference group.

*Abbreviations: BMIp, body mass index percentile; BP, blood pressure; pct, percentile; pDBP, peripheral diastolic blood pressure; cDBP, central diastolic blood pressure.*
